# Characterization of triclosan metabolism in *Sphingomonas* sp. strain YL-JM2C

**DOI:** 10.1038/srep21965

**Published:** 2016-02-25

**Authors:** Sikandar I. Mulla, Han Wang, Qian Sun, Anyi Hu, Chang-Ping Yu

**Affiliations:** 1 Key Laboratory of Urban Pollutant Conversion, Institute of Urban Environment, Chinese Academy of Sciences, Xiamen, 361021 China; 2 College of Ecology and Resource Engineering, Wuyi University, Wuyishan City 354300, China

## Abstract

Triclosan (TCS) is one of the most widespread emerging contaminants and has adverse impact on aquatic ecosystem, yet little is known about its complete biodegradation mechanism in bacteria. *Sphingomonas* sp, strain YL-JM2C, isolated from activated sludge of a wastewater treatment plant, was very effective on degrading TCS. Response surface methodology (RSM) was applied to optimize the conditions like temperature and pH. From RSM, the optimal TCS degradation conditions were found to be 30 °C and pH 7.0. Under optimal conditions, strain YL-JM2C completely mineralized TCS (5 mg L^−1^) within 72 h. Gas chromatography-mass spectrometry analysis revealed that 2,4-dichlorophenol, 2-chlorohydroquinone and hydroquinone are three main by-products of TCS. Furthermore, stable isotope experimental results revealed that the ^13^C_12_-TCS was completely mineralized into CO_2_ and part of heavier carbon (^13^C) of labeled TCS was utilized by strain YL-JM2C to synthesize fatty acids (PLFAs). Cell surface hydrophobicity (CSH) and degradation test results suggested that the strain could enhance degradation capacity of TCS through increasing CSH. In addition, the bacterium also completely degraded spiked TCS (5 mg L^−1^) in wastewater collected from the wastewater treatment plant. Hence, these results suggest that the strain has potential to remediate TCS in the environment.

Triclosan [5-chloro-2-(2,4-dichlorophenoxy)-phenol, TCS], also known as Ingrasan, is a biocide that inhibits the enoyl-acyl-carrier protein-reductase, which is an essential enzyme for the synthesis of fatty acids to develop bacterial cells^1^. Due to its strong inhibition activity against most of the bacteria, it has been widely used in many synthetic products like textiles, plastics, deodorants, soaps, toothpastes, etc^2^. Therefore, TCS can be released during usage and enter the sewage system and reach the wastewater treatment plant (WWTP). In WWTPs, TCS could only be partially removed and the removal efficiency varies significantly among different plants. Because of the effluent discharge and/or leaching from biosolids applied to agricultural fields, TCS is constantly released into the ecosystem^3^. As a result, TCS has been frequently detected with its by-products in the environmental matrices like ground water, wastewater, sediments, river, and sea^1^. For example, in the southeastern part of China, TCS was detected in influent as well as effluent of the WWTP located in Xiamen and was also detected in all of the 31 sampling sites in Jiulong river and its estuary in Fujian province^4^,^5^.

Studies suggest that TCS has weak androgenic activity against aquatic organisms and revealed both androgenic as well as estrogenic responses in human breast cancer cells^6^,^7^. Even under short term exposure to low concentration of TCS (environmentally relevant levels), fathead minnow gut microbiome was rapidly and significantly affected along with distorted^8^. In the environment, TCS might be transformed into chlorodioxins and dibenzofurans, when it was exposed to UV radiations or heat, and these transformed by-products are highly toxic than the parent compound^9^. Hence, researchers have raised great concerns over its potential adverse effects on environment. Since TCS is considered as an emerging contaminant, researchers have employed various chemical treatment methods for its degradation^10^,^11^,^12^. However, incomplete degradation of TCS in the chemical treatment could result in the production of toxic by- products like chlorophenoxy-phenols, chlorophenols, trihalomethanes, and dioxins, which are known to be carcinogenic^13^,^14^,^15^,^16^,^17^,^18^.

Biodegradation of triclosan in the environment and wastewater has recently attracted more attention. To date, some researches have used the stable isotope probing by ^13^C-labeled TCS to aid for tracking the movement of the heavier carbon (^13^C) from the labeled compound into the DNA (genetic biomarker) of the active TCS-utilizing microorganisms in the microbial consortia^1^,^3^. However, for the isolated TCS-degrading bacteria, there was not enough conclusive evidence to support that bacteria could utilize TCS for biosynthesis. Though two soil bacteria *Pseudomonas putida* and *Alcaligenes xylosoxidans* subsp. *denitrificans* were reported to grow on the TCS-containing agar plates^19^, most of the isolated TCS-degraders such as *Sphingomonas* sp. Rd1, *Nitromonas europaea*, *Sphingomonas* sp. PH-07 and S*phingopyxis* strain KCY1 were not shown to use TCS as a sole source of carbon^9^,^20^,^21^,^22^. The bacterial cell surface hydrophobicity (CSH) is a major parameter that governs bacterial adhesion, uptake and degradation of organic pollutants^23^. TCS is a hydrophobic antibacterial drug but the relationships between CSH and TCS degradation is still unclear. In bacteria, TCS was transformed into various by-products such as monohydroxy-TCS, dihydroxy-TCS, 2,4-dichlorophenol, 4-chlorophenol, 4-chlorocatechol, 3,5-dichlorocatechol, phenol, 2-methoxy-3,5-dichlorophenol, catechol and 2-chlorohydroquinone^21^,^24^,^25^, some of which are also potential carcinogens. Therefore, understanding the TCS degradation mechanism and pathway in different wastewater bacteria are critical for enhancing effective biodegradation of TCS in wastewater.

In this study, we isolated and identified TCS-degrading bacterium strain YL-JM2C and optimized conditions for TCS degradation by Response surface methodology (RSM). We demonstrated that strain YL-JM2C could utilize TCS as the carbon source by analyzing the flow of heavier carbon (^13^C) from ^13^C_12_-TCS into bacterial cellular fatty acids (phospholipid fatty acids, PLFAs). The changes of the bacterial CSH during biodegradation of TCS were also studied to provide a theoretical basis for the bacterial interaction of emerging contaminants like TCS in the ecosystem. In the end, the aim of this study was to address the unanswered issues for TCS biodegradation.

## Results

### Identification of a TCS-degrading bacterium

**Figure 1:**
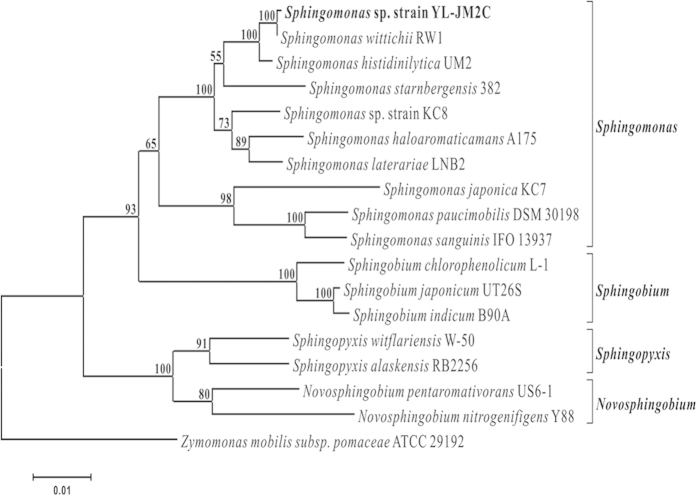
A maximum-likelihood phylogenetic tree showing the position of *Sphingomonas* sp. strain YL-JM2C relative to other type strains within the genus *Sphingomonas*. Building of the tree also involves a bootstrapping process repeated 100 times to generate a majority consensus tree. *Zymomonas mobilis* subsp. *pomaceae* served as the outgroup for the analysis. The scale bar indicates the number of substitutions/site.

A TCS-degrading bacterial strain YL-JM2C was isolated from a WWTP, Xiamen, China. The bacterium was identified as *Sphingomonas* sp. strain YL-JM2C on the basis of 16 S rRNA gene sequence analysis and its phylogenetic relationship with other bacteria was shown in Fig. 1. The detailed description of the cell morphology and biochemical characterization of the bacterium was provided in Supplementary Information (SI). Structural morphology of strain YL-JM2C was observed by scanning electron microscope (Figure S1 in SI). The draft genome sequence of *Sphingomonas* sp. strain YL-JM2C was submitted to the GenBank under accession number ASTM01000000.

### Optimization of growth and degradation studies

RSM based on Box Behnken design was applied to investigate the main and interactive effects of significant parameters including temperature (X_1_), pH (X_2_) and biomass (X_3_) on TCS degradation by strain YL-JM2C. Design established conditions of variables collectively with the experimental responses are given in Table 1. SAS software containing Response surface regression procedure was used to assess the data (Table 1) with some of non-significant interaction coefficients (*p* < 0.05) eliminated. The following equation was obtained to clarify the TCS degradation in strain YL-JM2C.


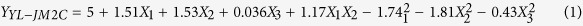


**Figure 2:**
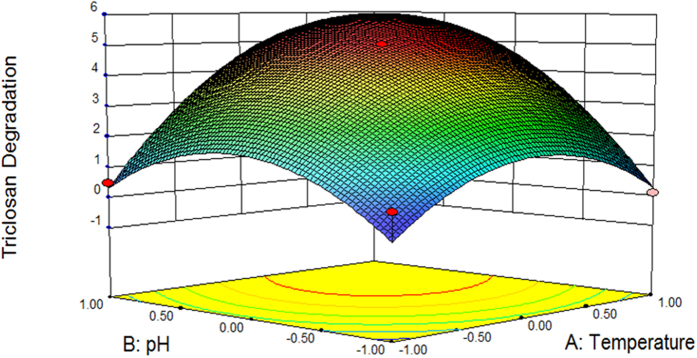
Three-dimensional plot of RSM showing the effects of temperature and pH on the degradation of TCS (5 mg L^−1^) by strain YL-JM2C with biomass (0.2 g L^−1^). Values are means ± standard deviations of three replicates.

*Y*_*YL*−*JM2C*_ is the predicted TCS degradation by strain YL-JM2C. X_1_, X_2_, and X_3_ are the coded values of temperature, pH and biomass, respectively. An R^2^ of 0.9527 indicated that 95.27% of the randomness in response could be covered by the model, signifying that the predicted values of the model were in agreement with the experimental values. The results of regression analysis showed that the linear term coefficient of temperature (X_1_) as well as pH (X_2_), the square term coefficient of temperature (X_1_) as well as pH (X_2_) and the cross-product term coefficient of temperature (X_1_) and pH (X_2_) showed significant effects (*p* < 0.05) on the TCS degradation by strain YL-JM2C whereas the linear term coefficient of biomass (X_3_) and square term coefficient of biomass (X_3_) were not significant (*p* > 0.05). As the value of inoculum biomass (non-significant variable) fixed at 0.2 (g L^−1^), the three dimensional response surface was plotted to directly show the effects of temperature and pH on TCS degradation in strain YL-JM2C (Fig. 2). As shown in Fig. 2, the plot of TCS degradation by strain YL-JM2C had a theoretical maximum value 5 mg L^−1^ at the stationary point. At the stationary point, the optimum levels for the two parameters of X_1_ and X_2_ were found to be 0.000 and 0.000 in terms of the coded units, i.e., temperature 30 °C and pH 7.00, respectively. Hence, the optimal conditions for TCS degradation in strain YL-JM2C were found to be 30 °C and pH 7.00 with inoculum biomass at 0.2 (g L^−1^).

**Figure 3:**
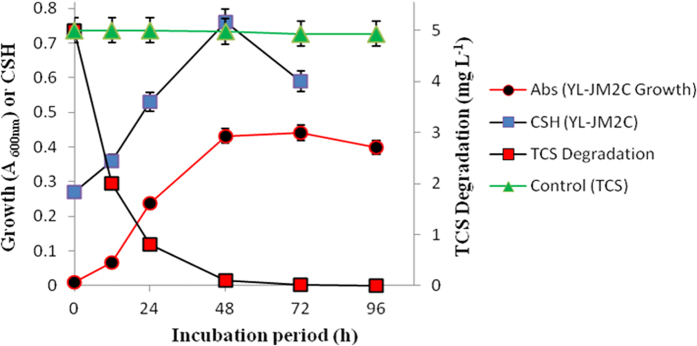
Bacterial growth and CSH of strain YL-JM2C during degradation of TCS (5 mg L^−1^). Values are means ± standard deviations of three replicates.

For growth studies, *Sphingomonas* sp. strain YL-JM2C was grown on yeast extract (0.04%)-containing ammonium mineral salts medium (AMS) with TCS (5 mg L^−1^). The growth of the strain was monitored on spectrophotometer with the increase in optical density at 600 nm and the maximum absorbance of growth was recorded as 0.44. The strain degraded TCS completely within 72 h (Fig. 3) with the corresponding release of chloride ions (Fig. S2). The bacterial strain degraded TCS when the range of the concentration was from 4.0 to 7.0 mg L^−1^ (Fig. 3 and Figure S3) and was able to degrade up to 99% of TCS at a given concentration of 7 mg L^−1^. However, when TCS concentration was 7 mg L^−1^, the growth of strain YL-JM2C was somewhat inhibited since the maximum absorbance of growth was around 0.32, which is less than 0.44 for 5 mg L^−1^ TCS.

### Hydrophobicity of strain YL-JM2C during degradation of TCS

The bacterial degradation of TCS carried out under optimal condition obtained by RSM and the CSH against TCS degradation with respect to incubation period were plotted (Fig. 3). Initially during degradation of TCS, the bacterial CSH increased quickly and subsequently reached its maximum hydrophobicity of 0.76 within 48 h (Fig. 3), and in the meantime bacterial growth and TCS degradation reached maximum. After the complete degradation of TCS in the medium, the CSH of strain YL-JM2C declined rapidly.

### Identification of metabolites

**Figure 4:**
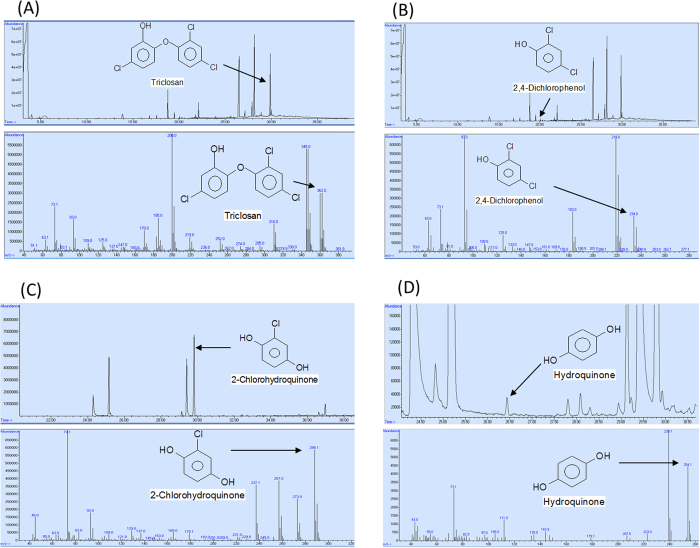
GC-MS analysis of the metabolites produced from TCS degradation by *Sphingomonas* sp. strain YL-JM2C. The retention times of the compounds were 28.106 (m/z, 362), 19.487 (m/z, 234), 29.822 (m/z, 288) and 26.440 (m/z, 254) min, respectively, which were identified as TCS-TMS, 2,4-dichlorophenol-TMS, 2-chlorohydroquinone-TMS and hydroquinone-TMS. TCS-TMS and 2,4-dichlorophenol-TMS were identified and compared by reported data^9^,^25^ whereas 2-chlorohydroquinone-TMS and hydroquinone-TMS were identified by their authentic compounds (Provided in supplementary information Figure S4).

To elucidate the pathway of TCS degradation by *Sphingomonas* sp. strain YL-JM2C, TCS intermediates were extracted and confirmed by gas chromatography-mass spectrometry (GC-MS). The degradation by-products identified on the basis of retention time with respect to their mass spectrum were matched with authentic standard compounds and also reported data. The peak at retention time of 28.160 min corresponded to trimethylsilyl (TMS)-TCS and decreased over time and completely disappeared within 84 h. TCS disappeared with the release of three major intermediates (Fig. 4). In 0 h sample, a peak of TMS-TCS was detected with the retention time 28.106 min. In the sample of 8 h, the reduction of TMS-TCS was observed along with a peak of first metabolite at 19.487 min (retention time) (Fig. 4A,B). In the 54 h sample, the peak of the first metabolite disappeared with the occurrence of two new peaks (the second and third metabolites) at 29.822 and 26.440 min (retention time) (Fig. 4C,D), respectively and TCS was detected in decreasing quantities. On the basis of these results, the presence of three by-products of TCS biodegradation were identified as 2,4-dichlorophenol (first metabolite), 2-chlorohydroquinone (second metabolite) and hydroquinone (third metabolite) with their authentic compounds (Figure S4) as well as the previously reported data^9^,^25^.

### Enzyme assay for chlorohydroquinone dehydrogenase

In the crude extract of the TCS induced cells of strain YL-JM2C, we have detected enzyme activity for chlorohydroquinone dehalogenase. During the enzyme assay we observed stoichiometric release of chloride ions (data not shown), which suggested the conversion of 2-chlorohydroquinone to hydroquinone. GC-MS analysis of the sample confirmed the formation of hydroquinone.

### Tracking experiment studied by ^13^C_12_-TCS

**Figure 5:**
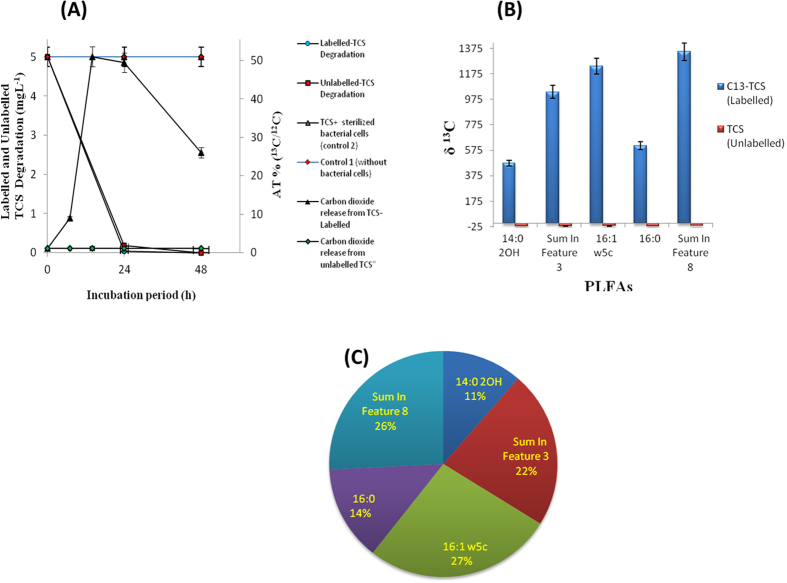
Results of ^13^C_12_-TCS labeling study in *Sphingomonas* sp. strain Yl-JM2C. (**A**) Release of carbon dioxide during degradation of labeled and unlabeled TCS; (**B**) the δ values of PLFAs; (**C**) distribution of assimilated ^13^C in individual PLFAs. Values are means ± standard deviations of three replicates.

In the tracking experiment using ^13^C_12_-TCS, the percentage of ^13^C/^12^C of the headspace carbon dioxide (CO_2_) rapidly increased to about 51% within 24 h. Yet, in unlabeled TCS, the percentage of ^13^C/^12^C of the headspace CO_2_ remained low around 1% (Fig. 5A). Hence, the results suggest that the strain could completely mineralize the given concentration of TCS into CO_2_. In addition, we also studied whether the bacterium would also utilize TCS as a carbon source for anabolism. To clarify the ambiguity, the PLFAs of strain YL-JM2C were also extracted and analyzed by stable isotope ratio mass spectrometer (SIRMS) after incubation with ^13^C_12_-TCS in the microcosm. Results of δ^13^C of PLFAs showed that the PLFAs of 14:0 2OH, 16:0, sum in feature 3, sum in feature 8 and 16:1 w5c have contained significant quantity of heavier carbon (^13^C) from labeled TCS (Fig. 5B). From these results, strain YL-JM2C not only mineralized TCS, the microorganism also synthesized its fatty acids by utilizing TCS as a sole source of carbon. Further, we had estimated the percentage of ^13^C assimilated into bacterial PLFAs. Analytical result indicated that strain YL-JM2C incorporated 61.58% of added ^13^C into PLFAs and the incorporation proportions of ^13^C were about 6.99%, 8.37%, 13.84%, 15.9% and 16.48% for the PLFAs of 14:0 2OH, 16:0, sum in feature 3, sum in feature 8 and 16:1 w5c, respectively. Moreover, the distribution of assimilated ^13^C was approximately 11%, 14%, 22%, 26% and 27% for the PLFAs of 14:0 2OH, 16:0, sum in feature 3, sum in feature 8 and 16:1 w5c (Fig. 5C). Incorporation percentages were related to the content of individual PLFA in the bacteria.

### Microcosm studies

In order to determine the ability of *Sphingomonas* sp. strain YL-JM2C to degrade TCS in wastewater, we performed microcosm studies by sterile influent wastewater, non sterile influent wastewater and non sterile effluent wastewater. *Sphingomonas* sp. strain YL-JM2C completely degraded TCS in the test microcosm with sterile and non sterile influent wastewater within 24 h (Figure S5A,B). In another test microcosm with non sterile effluent wastewater, the complete TCS depletion occurred also within 24 h (Figure S5C). In controls with sterile as well as non sterile influent wastewater and non sterile effluent wastewater, no degradation was observed even after 72 h (Figure S5).

## Discussion

**Figure 6:**
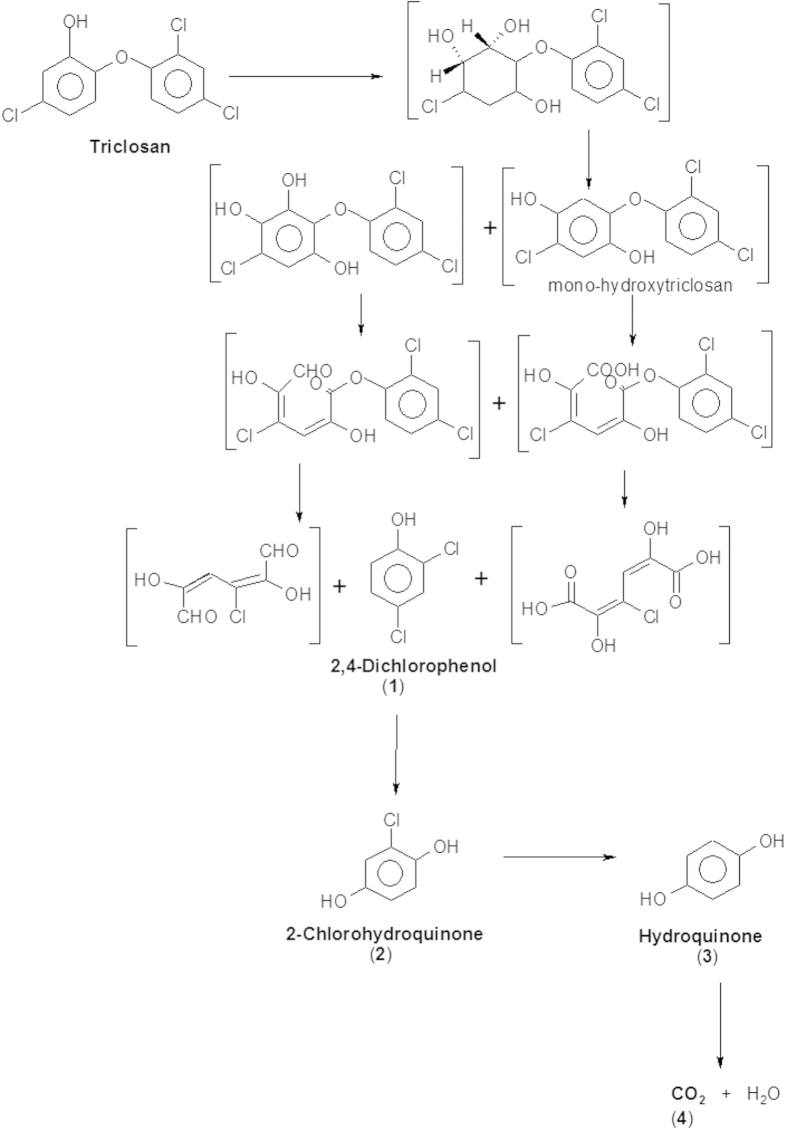
A proposed pathway for the degradation of TCS by *Sphingomonas* sp. strain YL-JM2C. The compounds shown in the brackets are hypothetical metabolites.

A TCS-degrading bacterium was identified as a *Sphingomonas* sp. strain YL-JM2C and characterized its ability to degrade TCS under different parameters. Previously, many studies have shown that the pH and temperature are the two important variables concerned and strongly influence the degradation of toxic pollutants by microorganisms^23^,^26^,^27^,^28^,^29^. RSM is an efficient method and has been successfully applied to get optimized conditions for different microorganisms involved in the degradation of xenobiotics^23^,^27^,^30^,^31^. Hence, it is used in this study to identify optimal conditions for TCS degradation in strain YL-JM2C. The results of our study indicated that strain YL-JM2C degraded TCS at different temperature (20–40 °C) and pH (5–9). In the present study we have developed a standard mathematical model, which could be efficiently used to predict and optimize conditions for the degradation of TCS in strain YL-JM2C within a given parameters. The bacterium could effectively degrade 5 mg L^−1^ of TCS (Fig. 3) under its optimized conditions. The bacterium was also able to degrade up to 99% of TCS at a given concentration of 7 mg L^−1^ (Figure S3). 2,4-Dichlorophenol, 2-chlorohydroquinone and hydroquinone are identified as major metabolites of the TCS degradation pathway in strain YL-JM2C (Fig. 6). This is the first report of the formation of hydroquinone in the degradation pathway of TCS. The initial mechanism of degradation of TCS by strain YL-JM2C was similar to other microorganisms^9^,^21^,^25^. In strain YL-JM2C, TCS was oxidatively transformed into 2,4-dichlorophenol by breaking ether bond present between two aromatic rings of TCS similar to other bacteria like strain PH-07, strain KCY1 and strain RHA1. The bacterium draft genome of strain YL-JM2C has 45 monooxygenase and 101 dioxygenase enzymes^32^ which are probably involved in these reactions^9^,^25^. TCS was also transformed to 2,4-dichlorophenol in fungus like *Trametes versicolor* and *Aspergillus versicolor*^33^,^34^. 2,4-Dichlorophenol was further transformed into 2-chlorohydroquinone by dechlorination. Similar result was observed in strain RHA1 whereas in strain PH-07, 2,4-Dichlorophenol was transformed into 3,5-dichlorocatechol and 4-chlorocatechol via two different routes. In this study, 2-chlorohydroquinone was dechlorinated and formed hydroquinone. Similar pathway was observed in strain RKJ 800^35^ during the degradation of 2-chloro-4-nitrophenol. Finally, this non-chlorinated compound hydroquinone was transformed into CO_2_ (Fig. 6).

The results of CSH indicated that the hydrophobicity of strain YL-JM2C might be associated with the concentration and toxic effect of TCS. Several research groups reported that the higher CSH was potentially useful in pesticide as well as petroleum bioremediation and survival for bacteria in the environment^23^,^36^. CSH is one of the most important factors that govern bacterial adhesion, uptake and degradation of hydrophobic organic compounds. Previous study has shown that high CSH of strains could enhance the degradation of hydrophobic pesticides^23^. However, there is no report on the relationship between high CSH and degradation ability of TCS which is also a hydrophobic compound. In our study we found that the presence of TCS could increase the hydrophobicity of strain YL-JM2C. As shown in the Fig. 3, with the increasing CSH of the strain YL-JM2C, bacterial growth as well as the TCS degradation increased. When the CSH maintained its maximum level at 48 h, the bacterial growth and TCS degradation efficiency reached their highest level. After the complete degradation of TCS, the CSH of strain YL-JM2C decreased rapidly. These results suggested that the CSH which governed adherence to the surface of the hydrophobic compounds could help the capability of the strain YL-JM2C to absorb and degrade TCS. Therefore, the strain with high hydrophobicity could be potentially useful for the remediation of wastewater containing hydrophobic emerging contaminants such as TCS.

In strain YL-JM2C, to confirm complete mineralization of TCS and also the use of TCS as a sole source of carbon for anabolism, stable isotope labeled TCS (^13^C_12_-TCS) was used and no yeast extract was added throughout the experiment. By the determination of heavier carbon (^13^C) released from labeled TCS into CO_2_, we can confirm TCS is completely mineralized by strain YL-JM2C into CO_2_. In addition, since fatty acids are necessary for building cell membrane of living microorganisms, we have also confirmed that the ^13^C from labeled TCS was used by strain YL-JM2C to synthesize its cellular fatty acids. These results suggest that the bacterium is not only able to mineralize TCS into CO_2_ but also utilize TCS as a sole source of carbon for its biosynthesis. Due to the low solubility and antimicrobial activity of TCS, we only used 4–7 mg L^−1^ TCS in the medium, and this low concentration of carbon source was unable to cause significant bacterial growth observed using optical density at 600 nm (data not shown). Yeast extract was added to help strain YL-JM2C to show more significant growth in the presence of TCS. The stable isotope result demonstrated that strain YL-JM2C could mineralize and utilize TCS for biosynthesis in the absence of yeast extract. In addition, TCS was used to block an enzyme called NADH dependent enoyl-acyl carrier protein reductase (encoded by *fabI* gene), which blocks the synthesis of fatty acids in the bacteria. In this study, stable isotope experiment of PLFAs demonstrated that strain YL-JM2C could assimilate the carbons located in TCS into fatty acids like 14:0 2OH, 16:0, sum in feature 3, sum in feature 8 and 16:1 w5c. This result suggested that strain YL-JM2C has novel mechanism to overcome the antimicrobial activity of TCS. Future studies are needed to quantify the amount of ^13^C-TCS transformed into each of its by-products, calculate the concentration of ^13^C-TCS entering into bacterial cell biomolecules, and also identification of responsible genes for the resistance and degradation of TCS in *Sphingomonas* sp. strain YL-JM2C.

## Conclusion

In conclusion, *Sphingomonas* sp. strain YL-JM2C isolated in the present study appeared to be efficient in degrading TCS. RSM was successfully applied to optimize the conditions for TCS degradation by strain YL-JM2C. Three different metabolites, 2,4-Dichlorophenol, 2-chlorohydroquinone and hydroquinone, were detected during degradation of TCS. Our results demonstrated that ^13^C_12_-TCS was completely mineralized into CO_2_ and the heavier carbon (^13^C) of labeled TCS was utilized by strain YL-JM2C for fatty acids synthesis. The CSH of strain YL-JM2C increased during the degradation of TCS and the increasing CSH helped the ability of strain YL-JM2C to degrade TCS. Overall, this study suggested that strain YL-JM2C could be a good candidate for the remediation of TCS in the contaminated wastewater.

## Materials and Methods

### Chemicals and culture media

Irgasan (Triclosan, TCS; 97% HPLC Grade) was purchased from Sigma-Aldrich Co., USA. Triclosan (^13^C_12_, 99%) was purchased from Cambridge Isotope Laboratories, Inc, USA. Acetone, acetonitrile and methanol were purchased from Merck, Germany. All other chemicals were of pure analytical-grade or highest grade available commercially. Stock solution (5 g L^−1^) of TCS was prepared with acetone and stored in brown bottles at −20 °C before use. AMS composition: K_2_SO_4_, 0.98 mM; KH_2_PO_4_, 3.9 mM; Na_2_HPO_4_.12 H_2_O, 6.1 mM; (NH_4_)_2_SO_4_, 5.88 mM; MgSO_4_.7 H_2_O, 0.15 mM; CaSO_4_.2 H_2_O, 0.07 mM; CoMoO_4_, 0.004 mM; KI, 0.001 mM; ZnSO_4_.7 H_2_O, 0.002 mM; MnSO_4_.H_2_O, 0.002 mM; H_3_BO_3_, 0.002 mM; FeSO_4_.H_2_O, 0.08 mM; H_2_SO_4_, 0.1 mM. The AMS medium was set to pH 7.00 (using 2 M NaOH or 2 M H_2_SO_4_) and sterilized in autoclave at 121 °C.

### Isolation and genome sequencing of *Sphingomonas* sp. strain YL-JM2C

*Sphingomonas* sp. strain YL-JM2C was isolated from activated sludge in a WWTP, Xiamen (China), through enrichment on TCS. For enrichment, 5 mL of activated sludge was added to 250 mL Erlenmeyer flask containing 95 mL of AMS with TCS (5 mg L^−1^) as a sole source of carbon (acetone free). The enrichment flask was incubated at 30 °C under dark condition on shaker at 150 rpm. Upon complete degradation, 5 mL of cultures were transferred to a new flask containing 95 mL of AMS with TCS (5 mg L^−1^). After 6 months of enrichment, one milliliter of the enrichment culture was serially diluted in R2A plates having TCS (5 mg L^−1^). Microorganisms grown on R2A agar plates containing TCS were further purified, and only one colony showed rapid degradation of TCS. The microorganism was designated as strain YL-JM2C. The microorganism was identified by using 16 S rRNA gene sequence analysis^37^. The whole genome sequence of YL-JM2C has been deposited in GenBank under accession number ASTM01000000^32^.

### Optimal condition, bacterial growth and TCS degradation

The experiments were conducted to elaborate important parameters like temperature, pH and biomass to get optimal condition for the degradation of TCS in strain YL-JM2C. In single-factor experiments, we studied the optimal ranges of three main variables such as temperature (20–40 °C), pH (5–9) and biomass (0.1–0.3 g L^−1^). RSM based on Box-Behnken design was used to optimize the above mentioned parameters and their interaction which significantly influenced TCS degradation in strain YL-JM2C^23^,^38^. Filter-sterilized yeast extract (0.04%) was added into AMS containing TCS (5 mg L^−1^). The microorganism was inoculated into the medium and the samples were collected after 72 h to quantify the residual TCS. A three-variable Box-Behnken design consisting of 15 experimental runs with 3 replicates at the midpoint was applied in this experiment. The experimental design was provided in Table 1 and the equation 2 shows the quadratic polynomial equation.


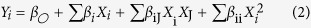


where Y_i_ is the predicted response, X_i_ and X_j_ are variables, β_o_ is the constant, β_i_ is the linear coefficient, β_ii_ is the quadratic coefficient and β_ij_ is the interaction coefficient.

To monitor the effects of TCS on the growth of strain YL-JM2C, strain YL-JM2C was grown in 500 mL Erlenmeyer flask containing 100 mL of yeast extract (0.04%)-containing AMS with appropriate concentration of TCS (4–7 mg L^−1^). The bacterial growth was measured at 600 nm by UV-spectrophotometer (UV-5200 Spectrophotometer). For degradation studies, the TCS degradation was monitored by HPLC. Uninoculated culture flasks with the same concentrations of TCS were served as a control.

### Identification of metabolites

The metabolic products of TCS in strain YL-JM2C cultures containing TCS (5 mg L^−1^) were extracted and identified by GC-MS. The samples were taken at regular intervals (0, 8, 24, 36, 54, 60, 72 and 84 h). The same bacterial culture supernatant lacking TCS was used as a negative control, and non inoculated control containing TCS (5 mg L^−1^) was included as well. The collected samples were centrifuged (7000 × *g*) for 20 min, adjusted to pH 2.0 with 2 M H_2_SO_4_ and were extracted with ethyl acetate. For extraction, supernatant was mixed with equal volume of ethyl acetate in a brown bottle and shaken for 6 h at 150 rpm on shaker. Further, the sample was transferred to separating funnel and allowed for 15 min to separate. The ethyl acetate layer was collected and the aqueous layer was used for one more time extraction with ethyl acetate. Both ethyl acetate layers were combined together and evaporated at room temperature on anhydrous sodium sulfate. The extracted samples were reconstituted in acetone (470 μL) and derivatized with BSTFA (30 μL). The derivatized samples were analyzed by the GC-MS. The metabolites identified by mass spectrometry analysis were matched with authentic standard compounds as well as reported data.

### Measurement of the relative CSH

The relative CSH of the strain YL-JM2C during the growth and biodegradation of TCS was assessed by the microbial adherence to hydrocarbon method^39^. The *p*-xylene (hydrocarbon) was used for CSH determination of *Sphingomonas* sp. strain YL-JM2C. The CSH value was calculated using equation 3.


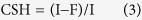


I and F represent the initial and the final optical densities at 600 nm of the aqueous phase.

### Enzyme assays

The cells of strain YL-JM2C grown on yeast extract (0.04%)-containing AMS medium supplemented with TCS (5 mg L^−1^) were harvested by centrifugation (8,000 × *g* for 10 min at 4 °C), washed and resuspended in 50 mM phosphate buffer (pH 7.0). The cell-free extract were prepared through ultrasonication (Ultrasonic homogenizer JY92-IIN, Ningbo Scientz Biotechnology Co., Ltd., Ningbo, China) followed by centrifugation (12,500 × *g* for 40 min at 4 °C). The supernatant obtained was used as the crude enzyme for enzyme assays^40^. Chlorohydroquinone dehalogenase activity was assayed according to the previous literature^35^. The enzyme activity was determined as the total chloride released at 30 °C. The reaction mixture contained 100 mM Tris-acetate buffer (pH 7.5), 0.2 mM NADPH, 5-7 mg of cell extract and 2-chlorohydroquinone (30 μM). The final volume of the reaction mixture was 5 mL and samples were collected at regular intervals for chloride ion analysis as described below. Protein content was determined by BCA protein assay kit (Thermo Scientific, USA).

### Stable Isotope Experiment

^13^C_12_-TCS was used to confirm TCS carbon utilization by the isolated bacterium strain YL-JM2C. Fifty milliliters of sterile ASM (without yeast extract) containing ^13^C_12_-TCS (5 mg L^−1^) was filled into a 120 mL serum bottle. Artificial air (nitrogen/oxygen, 21/79, v/v) was used to purge ASM to remove CO_2_. The serum bottle was then inoculated with strain YL-JM2C (optical densities at 600 nm = 0.25) and sealed by butyl rubber immediately. Meanwhile, unlabeled-TCS with the same concentration was also used in another set of bottles for comparison. Heat-killed culture (YL-JM2C) and uninoculated serum bottles, which were set up under identical conditions to those of the experimental cultures and served as controls. All treatments were incubated at 30 °C under dark conditions and kept on shaker at 150 rpm. ^13^C/^12^C ratio of the headspace CO_2_ was determined by SIRMS at 0, 7, 14, 25, and 48 h. Thereafter the bacterial cultures were harvested by centrifugation and washed twice with phosphate buffer. Bacterial fatty acids were extracted according to the manual of MIDI Sherlock Microbial Identification System and analyzed by SIRMS immediately. SIRMS conditions were described in the SI. The percentage of added ^13^C incorporated into a specific PLFA was calculated as: %^13^C incorporation = 100 × ((*F*_*labeled*_ − *F*_*unlabeled*_) × (PLFA*i*)_*labeled*_]/[^13^C added], with the concentrations of PLFA and ^13^C added in mg C kg^−1^ solution and *F* value as: *F* = ^13^C/(^13^C + ^12^C) = *R*/(*R* + 1). The carbon isotope ratio (*R*) was evaluated from δ^13^C values as: *R* = (δ^13^C/1000 + 1)*R*_VPDB_, with Pee Dee Belemnite standard *R*_VPDB_ = 0.0112^41^. The proportion of ^13^C incorporated into an individual PLFA of labeled PLFAs was expressed as: %^13^C incorporation = 100 × (^13^C_PLFA*i*_/Σ ^13^C_PLFA*i*)_, where ^13^C_PLFA*i*_ was the amount of ^13^C incorporated into the PLFA of “*i*”, which was calculated as: ^13^C_PLFA*i*_ = (*F*_*labeled*_ − *F*_*unlabeled*_) × [PLFA*i*]_*labeled*_^42^.

### Microcosm study for the degradation of TCS by strain YL-JM2C in wastewater

Wastewater samples were collected from raw sewage (influent) and treated wastewater (effluent) of a WWTP, Xiamen (China). The total nitrogen (TN), total phosphorus (TP) and total chemical oxygen demand (TCOD) of collected influent wastewater were 33.8, 2.5 and 95.6 mg L^−1^, respectively, whereas the TN, TP and TCOD of collected effluent wastewater were 15.6, 1.4 and 43.0 mg L^−1^, respectively. The pH of the samples was adjusted to pH 7.00 (using 1 M NaOH or 1 M H_2_SO_4_). For microcosm studies, the following four experiments with TCS (5 mg L^−1^) spiked wastewater were conducted: (I) test microcosm with sterile influent in which cells of strain YL-JM2C were inoculated (2.66 × 10^7^ CFU per 100 mL of wastewater) in the autoclaved influent; (II) test microcosm with non sterile influent in which cells of strain YL-JM2C were inoculated in natural influent; (III) test microcosm with non sterile effluent in which cells of strain YL-JM2C were inoculated in natural effluent; and (IV) heat-killed controls consisted of autoclaved cultures and non-inoculated controls without *Sphingomonas* sp. strain YL-JM2C, were set up under identical conditions to those of the experimental cultures at 30 °C.

### Analytical Methods

Metabolites of TCS in strain YL-JM2C were analyzed by GC-MS. TCS and ^13^C_12_-TCS residues at different intervals were determined by HPLC (Dionex Ultimate 3000, USA). Chloride ion analysis was carried out using Ion Chromatography. Detailed description of the analytical methods is provided in SI.

## Additional Information

**How to cite this article**: Mulla, S. I. *et al.* Characterization of triclosan metabolism in *Sphingomonas* sp. strain YL-JM2C. *Sci. Rep.* **6**, 21965; doi: 10.1038/srep21965 (2016).

## Supplementary Material

Supplementary Information

## Figures and Tables

**Table 1:** Box-Behnken experimental design and the response of dependent variable for TCS degradation in strain YL-JM2C.

Run	X_1_	X_2_	X_3_	Response
				[TCS Degradation (mg L^−1^) Y_YL−JM2C_]
1	0	0	0	5
2	0	−1	1	0.85
3	1	1	0	4.79
4	0	0	0	5
5	1	0	-1	4.84
6	0	1	1	4.72
7	−1	1	0	0.47
8	−1	0	−1	0.73
9	0	0	0	5
10	1	0	1	4.89
11	−1	−1	0	0.45
12	1	−1	0	0.11
13	0	1	−1	4.57
14	0	−1	−1	0.89
15	−1	0	1	0.86

Values are means ± standard deviations of three replicates.
