# Supplementary material for: Characterization of triclosan metabolism in Sphingomonas sp. strain YL-JM2C
Source: Sci Rep. 2016 Feb 25;6:21965. doi: 10.1038/srep21965 (PMC4766416; doi:10.1038/srep21965)
Supplement: Supplementary Information [file srep21965-s1.doc]

**Supplementary Information**

**Characterization of Triclosan Metabolism in *Sphingomonas* sp. strain YL-JM2C**

Sikandar I. Mulla,^1^ Han Wang,^2^ Qian Sun,^1^ Anyi Hu,^1^ and Chang-Ping Yu^1^*****

^1^Key Laboratory of Urban Pollutant Conversion, Institute of Urban Environment, Chinese Academy of Sciences, Xiamen, PR China.

^2^College of Ecology and Resource Engineering, Wuyi University, Wuyishan City 354300, PR China.

***Correspondence to:** Dr. Chang-Ping Yu,

Key Laboratory of Urban Environment and Health,

Institute of Urban Environment,

Chinese Academy of Sciences, Xiamen, China

Email: [cpyu@iue.ac.cn](mailto:cpyu@iue.ac.cn)

Phone No: +86- 592-6190768

Fax: +86-592-6190582

**Supporting Information: 8 pages, 5 figures**

**Materials and Methods**

**SIRMS procedure**

Thermo trace gas chromatography (USA) was implemented for SIRMS with the chromatographic column Rt®-Q-BOND (Restek, 30 m × 0.32 mm ID) for carbon dioxide and DB-5 (Agilent, 30 m × 0.25 mm ID) for PLFAs. The flow rates of carrier gas (He) were 1.8 and 1.2 ml min^-1^ for carbon dioxide and PLFAs, respectively. The temperature program was 40 °C for 6 min for carbon dioxide. For PLFAs, the temperature program was 100 °C for 2 min, and then ramping to 260 °C with a rate of 5 °C min^-1^. ^13^C value was determined by Thermo Delta V Advantage isotope ratio mass spectrometer (USA).

**Analytical Methods**

TCS concentrations were determined by HPLC (Dionex Ultimate 3000, USA) equipped with a UV detector (235 nm). The separation was performed on LabChrom C_18_ (5 µm, 4.6 mm × 250 mm, Hitachi) using methanol and water as the mobile phase at a flow rate of 1 ml min^-1^. The elution gradient was as follows: 83% methanol for 5 min and increased to 95% methanol up to 11 min, held at 95% methanol for 3 min, decreased back to 83% methanol over 0.5 min, and held at 83% methanol for 1.5 min. The limit of quantification was 0.1 mg L^-1^.

TCS degradation intermediates were analyzed by GC (Agilent 7890A)/MS (5975C) under full scan mode equipped with Agilent 19091S-433 HP-5MS capillary column (30 m × 250 µm × 0.25 μm, 5% phenyl methyl siloxane). The oven temperature was programmed from 60 ºC (10 min) to 300 ºC at 10 ºC min^-1^ followed by a 5 min hold at 300 ºC. The injector temperature was kept at 250 °C and 1 µl sample was injected. Helium was used as the carrier gas at a flow rate of 1.0 ml min^-1^. MS was operated under electron ionization mode at 70 eV with mass scan range of 40-800 amu.

Chloride analysis was carried out using an ion chromatography (IC 3000, Dionex) and an Ion Pac AG11-HC column (4 × 50 mm) using 30 mM KOH solution as an eluent with a flow rate of 1.0 ml min^-1^ at 30 °C. The measurement was carried out with a 20 µl sample and amount of chloride was quantitatively estimated by calibration curve of chloride standard (NaCl in water, National Center of Analysis and Testing for Nonferrous Metals and Electronic Materials). The detection limit for chloride ion was 0.1 mg L^-1^.

**Results**

**Identification of a TCS-degrading bacterium**

The activated sludge sample was enriched with TCS (5 mg L^-1^) as a sole carbon source and the strain YL-JM2C was obtained after numerous streaking on R2A agar plates[^1^](#_ENREF_1). The bacterial strain was aerobic, Gram-negative, rod-shaped (0.4-0.6 × 1.3-1.7 μm) (Figure S1), non-sporulating and non-motile. *Sphingomonas* sp. strain YL-JM2C was able to grow on R2A at 30-35 ºC. The bacterial strain was able to grow in different media such as nutrient broth, nutrient agar, R2A，R2A with TCS，R2A with triclocarban (TCC) and ammonium mineral salts (AMS) liquid medium with 0.04% yeast extract containing TCS (5 mg L^-1^) and TCC (4 mg L^-1^). The bacterium was able to grow on nutrient medium containing 1% of NaCl. The strain showed catalase activity and was able to reduce nitrate, assimilated decylic acid and phenyl acetic acid. The major cellular fatty acids of strain YL-JM2C are summed feature 8 (C_18:1 ω6c_ and C_18: 1 ω7C_) (50.29%), summed feature 3 (C_16:1 ω6c_ and C_16: 1 ω7c_) (14.41%), C_16:0_ (10.13%), C_14:0 2OH_ (6.75%) and C_19:0 cyclo ω8c_ (5.95%).


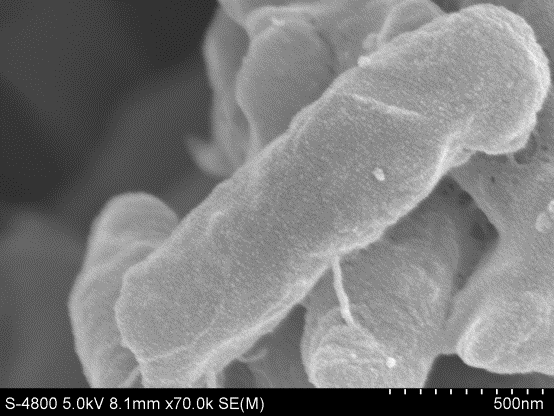

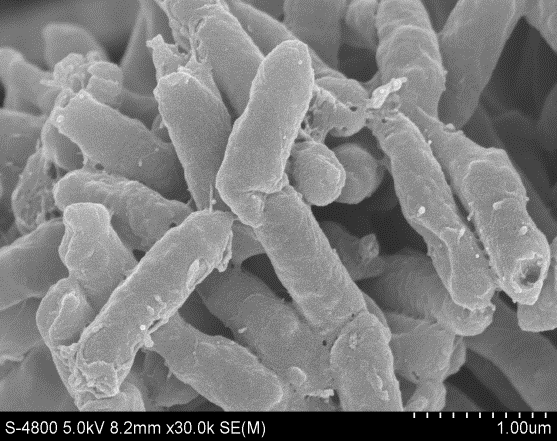


**Figure S1**. Scanning electron micrograph of *Sphingomonas* sp. strain YL-JM2C.


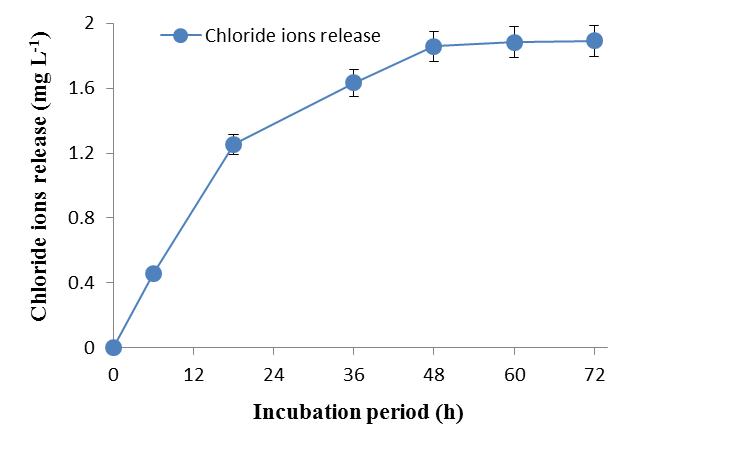


**Figure** **S2**. Release of chloride ions during degradation of TCS (5 mg L^-1^) in strain YL-JM2C. Values are means ± standard deviations of three replicates.

(A)
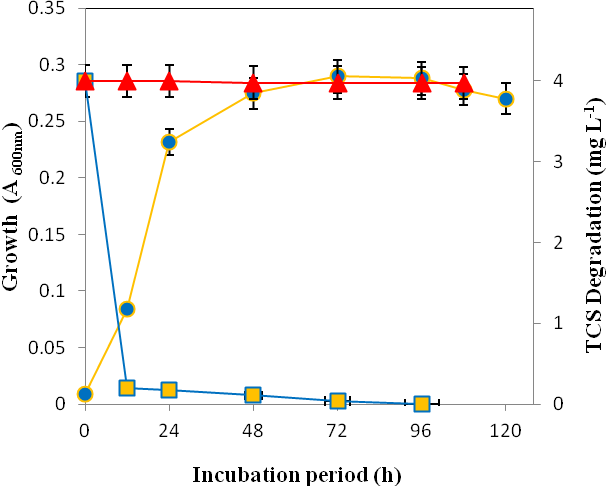


(B)
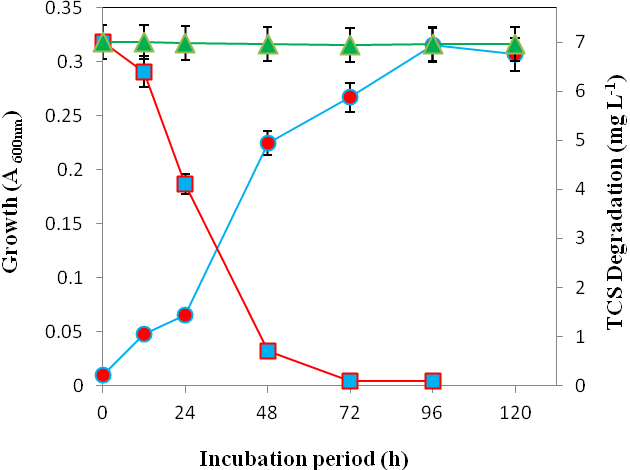


**Figure S3.** Degradation of various concentrations of TCS by strain YL-JM2C. Values are means ± standard deviations of three replicates.

(A)


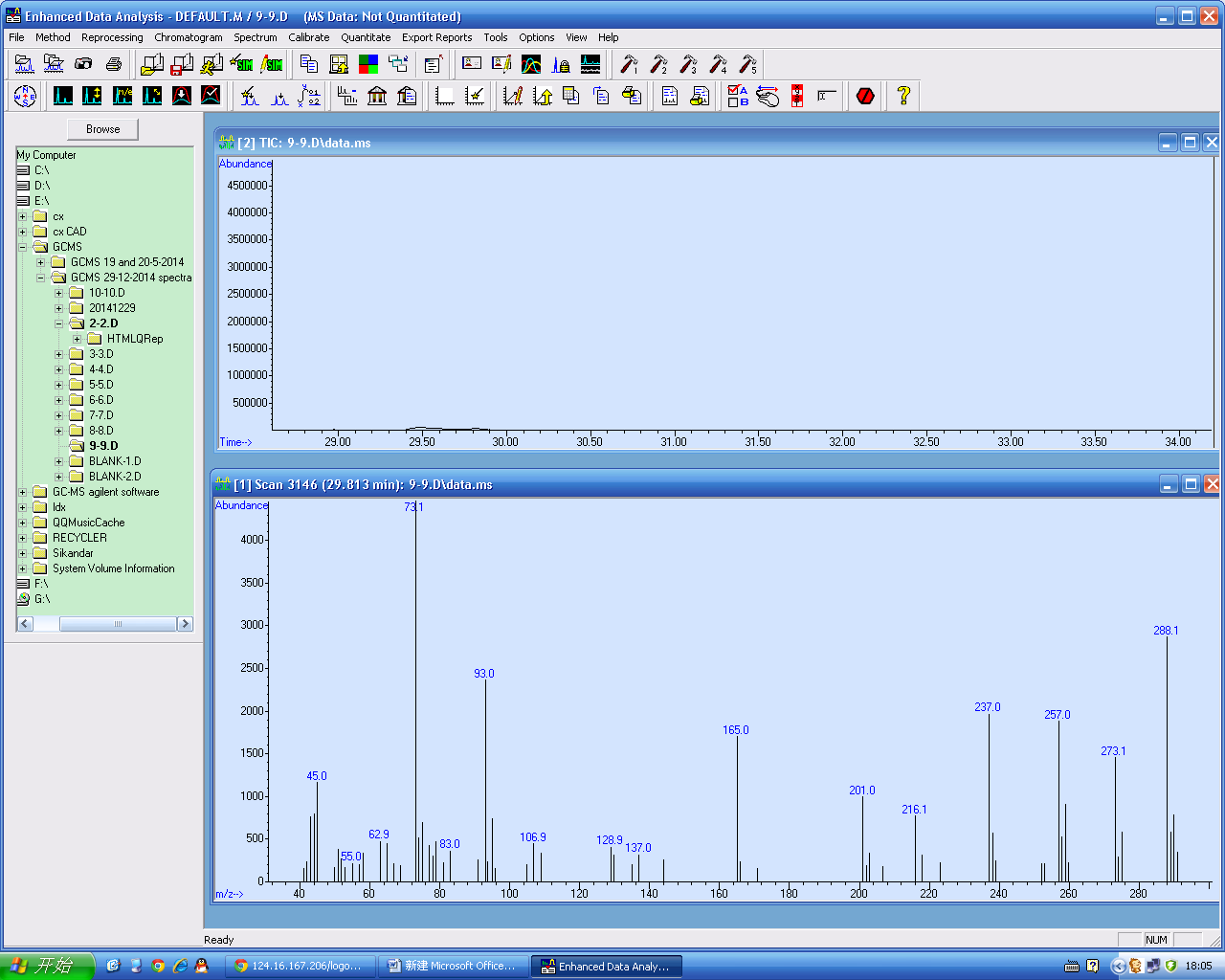

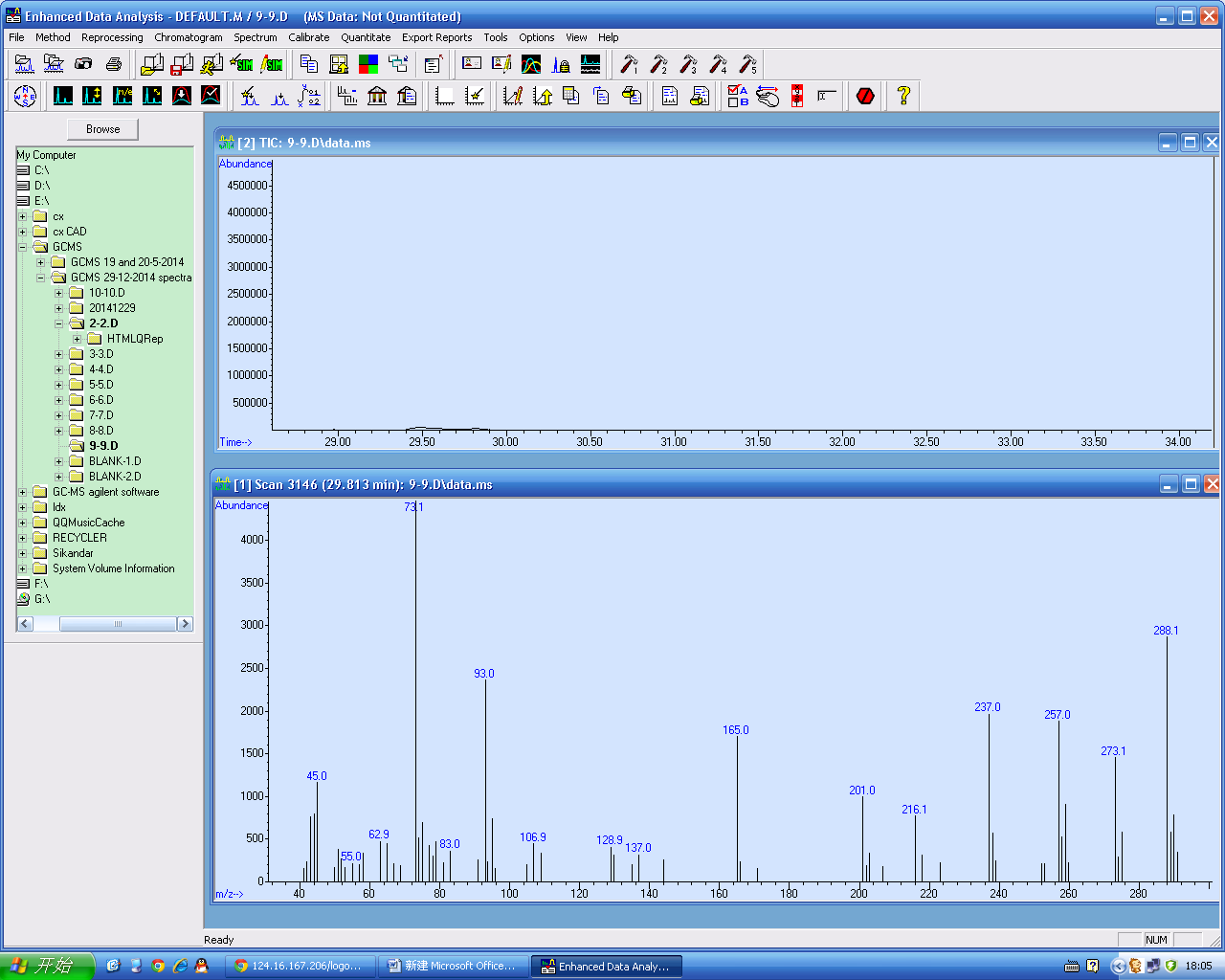


(B)


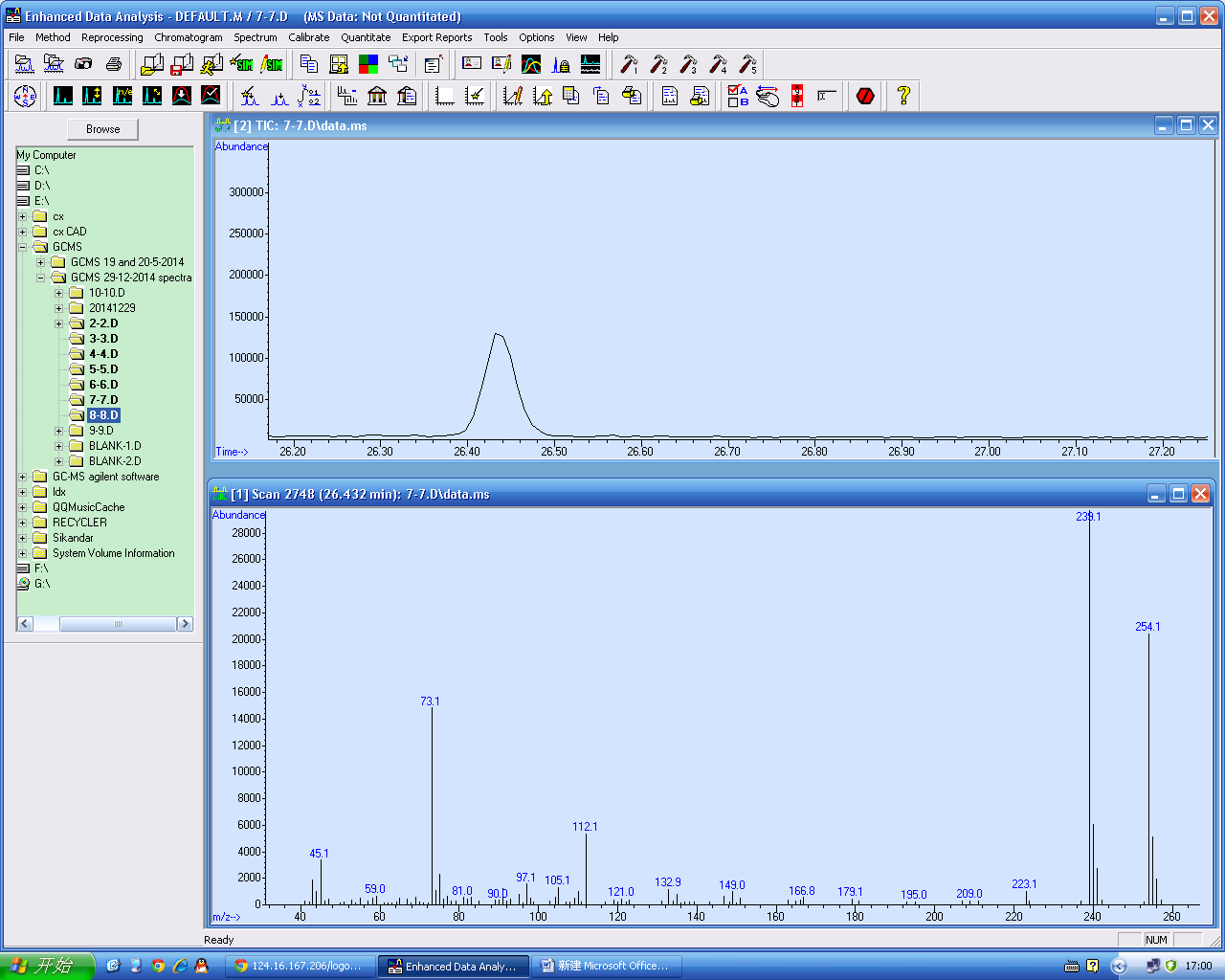


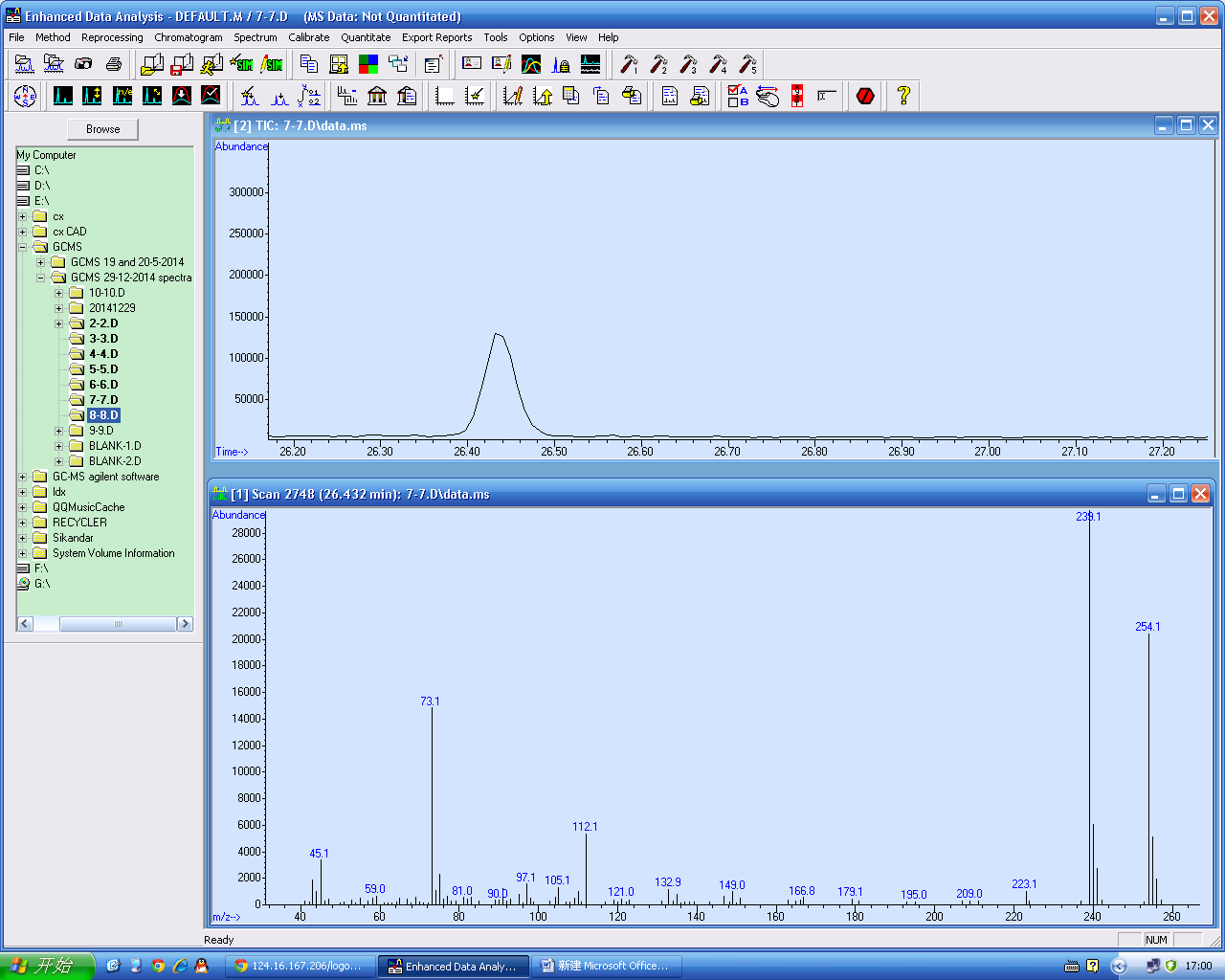


**Figure** **S4**. GC-MS spectra of authentic compounds, 2-chlorohydroquinone and hydroquinone.

(A)


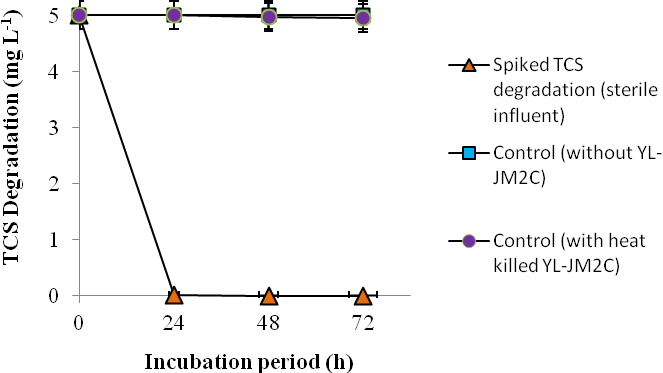


(B)


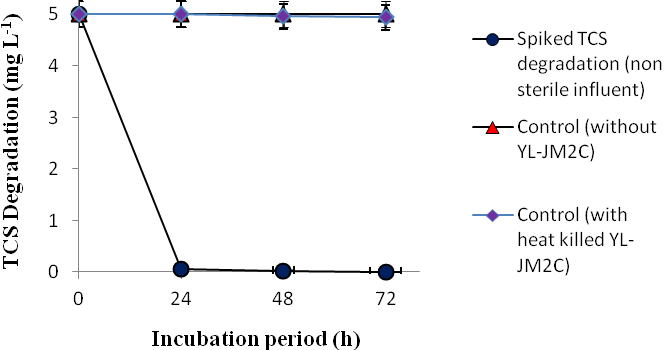


(C)


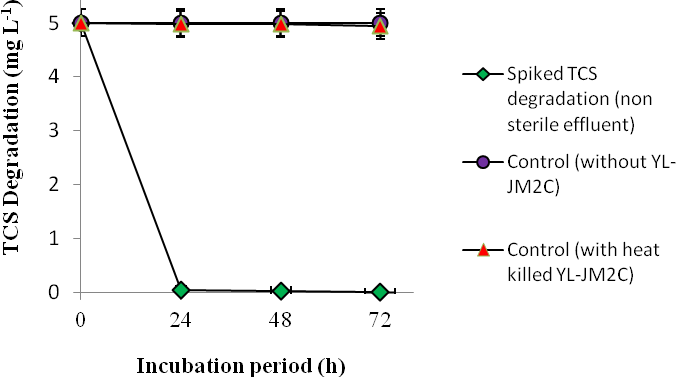


**Figure** **S5**. Degradation of spiked TCS (5 mg L^-1^) in wastewater by strain YL-JM2C. Values are means ± standard deviations of three replicates.

**References**

1. Reasoner, D. J. & Geldreich, E. E. A new medium for the enumeration and subculture of bacteria from potable water*. Applied and Environmental Microbiolo*g**y** 49, 1-7 (1985).
